# Tonic activation of GABA_B_ receptors via GAT-3 mediated GABA release reduces network activity in the developing somatosensory cortex in GAD67-GFP mice

**DOI:** 10.3389/fnsyn.2023.1198159

**Published:** 2023-05-30

**Authors:** Timo Ueberbach, Clara A. Simacek, Irmgard Tegeder, Sergei Kirischuk, Thomas Mittmann

**Affiliations:** ^1^Institute for Physiology, University Medical Center of the Johannes Gutenberg-University, Mainz, Germany; ^2^Institute of Clinical Pharmacology, Faculty of Medicine, Goethe-University, Frankfurt, Germany

**Keywords:** GABA, GABA_B_ receptors, somatosensory cortex, E/I balance, presynaptic, postsynaptic, GAT-3, neurodevelopmental disorders

## Abstract

The efficiency of neocortical information processing critically depends on the balance between the glutamatergic (excitatory, E) and GABAergic (inhibitory, I) synaptic transmission. A transient imbalance of the E/I-ratio during early development might lead to neuropsychiatric disorders later in life. The transgenic glutamic acid decarboxylase 67-green fluorescent protein (GAD67-GFP) mouse line (KI) was developed to selectively visualize GABAergic interneurons in the CNS. However, haplodeficiency of the GAD67 enzyme, the main GABA synthetizing enzyme in the brain, temporarily leads to a low GABA level in the developing brain of these animals. However, KI mice did not demonstrate any epileptic activity and only few and mild behavioral deficits. In the present study we investigated how the developing somatosensory cortex of KI-mice compensates the reduced GABA level to prevent brain hyperexcitability. Whole-cell patch clamp recordings from layer 2/3 pyramidal neurons at P14 and at P21 revealed a reduced frequency of miniature inhibitory postsynaptic currents (mIPSCs) in KI mice without any change in amplitude or kinetics. Interestingly, mEPSC frequencies were also decreased, while the E/I-ratio was nevertheless shifted toward excitation. Surprisingly, multi-electrode-recordings (MEA) from acute slices revealed a decreased spontaneous neuronal network activity in KI mice compared to wild-type (WT) littermates, pointing to a compensatory mechanism that prevents hyperexcitability. Blockade of GABA_B_ receptors (GABA_B_Rs) with CGP55845 strongly increased the frequency of mEPSCs in KI, but failed to affect mIPSCs in any genotype or age. It also induced a membrane depolarization in P14 KI, but not in P21 KI or WT mice. MEA recordings in presence of CGP55845 revealed comparable levels of network activity in both genotypes, indicating that tonically activated GABA_B_Rs balance neuronal activity in P14 KI cortex despite the reduced GABA levels. Blockade of GABA transporter 3 (GAT-3) reproduced the CGP55845 effects suggesting that tonic activation of GABA_B_Rs is mediated by ambient GABA released via GAT-3 operating in reverse mode. We conclude that GAT-3-mediated GABA release leads to tonic activation of both pre- and postsynaptic GABA_B_Rs and restricts neuronal excitability in the developing cortex to compensate for reduced neuronal GABA synthesis. Since GAT-3 is predominantly located in astrocytes, GAD67 haplodeficiency may potentially stimulate astrocytic GABA synthesis through GAD67-independent pathways.

## 1. Introduction

Gamma-aminobutyric acid (GABA), the main inhibitory neurotransmitter in the mature CNS, fulfills trophic functions during development (Meier et al., 1983; Represa and Ben-Ari, 2005) and gradual maturation of GABAergic system has been implicated in the timing of the critical period in sensory systems (for review, see Jiang et al., 2005). A reduced level of GABA can strongly impact brain functions both during development and in adulthood. Under healthy conditions the brain balances the strengths of glutamatergic excitation (E) vs. GABAergic inhibition (I), establishing an appropriate E/I balance. However, this E/I balance can be disturbed during development and throughout life. Numerous studies suggested that a transient E/I imbalance during early development cause structural and functional developmental malfunctions and manifests in behavioral deficits in adult rodents (for review, see Eichler and Meier, 2008; Ghatak et al., 2021). In humans those disbalanced states of the developing brain have been associated with neuropsychiatric diseases like autism-spectrum disorders (ASD) and schizophrenia, or promote epileptogenesis (Turrigiano and Nelson, 2004; Eichler and Meier, 2008; Naaijen et al., 2017; Bassetti et al., 2021; Kirischuk, 2022). However, we have previously observed an impaired GABAergic signaling in the contralateral cortical hemisphere in adolescent mice early after traumatic brain injury (TBI), resulting in a shift of the cortical E/I ratio toward excitation (Le Prieult et al., 2017). Such a trauma-induced E/I- imbalance was linked to the development of post traumatic epilepsy (Pavlov et al., 2011; Le Prieult et al., 2017; Dulla and Pitkanen, 2021; Ihbe et al., 2022). However, we found that expression of a presynaptic voltage-gated calcium channel (CaV1.3) in somatostatin-positive interneurons counterbalances the early impaired GABAergic inhibition and hyperactivity on the cortical network after TBI (Ihbe et al., 2022). In search for compensatory mechanisms, which can rebalance the E/I ratio in the cortex, the present study made use of the GAD67-GFP knock-in mouse line (KI), a well-established mouse model to study GABAergic interneurons in living tissues (Tamamaki et al., 2003). This mouse line significantly contributed to a better understanding of the role of GABAergic interneurons on normal brain development and also under pathological conditions like ASD, schizophrenia or TBI (Gandhi et al., 2008; Curley et al., 2013; Ihbe et al., 2022). However, the GFP labeling comes with the cost of one functional copy of the GAD1 Gen which codes the GAD67 glutamate decarboxylase enzyme, the main producer of GABA in the developing CNS (Erlander et al., 1991; Erlander and Tobin, 1991; Gonzales et al., 1991). As a consequence, the lack of one functional copy of the GAD1 gen leads to a reduced expression of the GAD67 enzyme and in turn a decreased GABA concentration in the CNS (Tamamaki et al., 2003). We hypothesized that the GABA shortage will shift the E/I ratio away from physiological equilibrium. Indeed, on the behavioral level several studies demonstrated changes in social behavior, like hyperactivity, ADHD-like behavior and changes in response to olfactory sensory stimuli in KI mice (Janitzky et al., 2009; Bruxel et al., 2016). However, the KI mouse line was not shown to generate more epileptic seizures compared to their wild type littermates (Tamamaki et al., 2003), which indicates potential compensatory mechanisms to prevent chronical hyperexcitability and epileptogenesis. Here we investigated the strength of the excitatory and inhibitory neurotransmission in layers 2/3 pyramidal neurons in the somatosensory cortex of young, developing KI-mice using electrophysiological patch-clamp recordings. In addition, we recorded cortical network activity in acute cortical slices using multi-electrode array (MEA) techniques. Surprisingly, although we observed reduced GABA levels and an elevated E/I ratio in the cortex of KI mice at P14, the MEA recordings disclosed a reduced neuronal network activity. To address the underlying mechanism(s), we assessed effects of blocking GABA_B_Rs or the GABA transporter 3 (GAT-3). The results suggest that the suppressed cortical network activity in the KI mice at P14 resulted from an inhibition of the glutamatergic excitatory transmission via tonically activated pre- and postsynaptic GABA-B-receptors (GABA_B_Rs), and that the ambient GABA required to activate those GABA_B_Rs was released through reverse operating GAT-3. Since GAT-3 is mainly expressed in astrocytes (Minelli et al., 1996) and was shown to have neuroprotective effects after brain trauma in rodents (Cho et al., 2022), we propose that low cortical GABA concentrations in young KI mice stimulates GABA efflux from astrocytes, thereby stabilizing neuronal network activity despite an observed GABA shortage.

## 2. Materials and methods

### 2.1. Animals and ethical statement

Juvenile, transgenic heterozygous GAD67-GFP positive (KI) mice (*n* = 41) and their GAD67-GFP negative (WT) littermates (*n* = 38) were used in two age groups on either postnatal days 14/15 (P14) or on the postnatal days 21/22 (P21).

This mouse line was originally generated by Tamamaki et al. (2003). For the present experiments we crossed KI- with wild-type C57BL/6N- mice. During preparation of the cortical tissue from the offspring (see section “2.3 Slice preparation”) each brain was optically inspected for presence or absence of GFP-positive cells. This was done by the experimenter in a non-blinded approach and by use of a fluorescent lamp (SFA Light Head, Nightsea, Hatfield, PA, USA) in the area of the cerebellum. Animals were kept under standard 12 h day/night rhythm at a constant temperature of 23^°^C with an *ad libitum* supply of food and water. The experiments were designed to restrict the number of animals that were used in this study to the necessary minimum, and all experiments were performed in accordance with German and European laws of animal welfare in science (2010/63/EU).

### 2.2. ELISA

The cortical GABA-concentration was examined by ELISA experiments using whole cortex lysates from KI and their WT littermates. Mice were deeply anesthetized with 4% isoflurane and decapitated. Next, the brains were quickly removed, the cortex isolated and frozen in liquid nitrogen. Cortex lysates were produced using an electrical homogenizer adding 1.5 ml N-PER Neuronal Protein extraction reagent (Thermo Fisher Scientific, Waltham, MA, USA) with the addition of the Halt Protease and Phosphatase Inhibition cocktail (1:100; Thermo Fisher Scientific, Waltham, MA, USA). After keeping the samples at 4^°^C for 1 h they were centrifuged at 13,000 rpm for 20 min at 4^°^C. The supernatant was used for further analysis. Samples were adjusted to a protein concentration of 1.5 mg/ml. The GABA concentration was measured with a GABA sandwich Elisa kit (Abcam, Cambridge, England). The fluorescence signals were measured using a plate reader (Infinite M1000, Tecan, Switzerland).

### 2.3. Slice preparation

Mice were deeply anesthetized with 4% isoflurane and decapitated. The brain was removed and transferred into ice cold oxygenated (95% O_2_ and 5% CO_2_) cutting artificial cerebrospinal fluid (cACSF) containing (in mM): 87 NaCl, 37.5 choline chloride, 2.5 KCl, 7MgSO_4_ × 7H_2_O, 0.5 CaCl_2_ × H_2_O, 1.25 NaH_2_PO_4_ × H_2_O, 25 NaHCO_3_, and 25 d-glucose; pH: 7.4 (ingredients purchased from Carl Roth, Karlsruhe, Germany).

Brains were cut into 350 μm coronal slices for patch-clamp experiments and 400 μm for MEA recordings using a vibratome (VT1200 S, Leica, Wetzlar, Germany). Brain slices containing the somatosensory cortex were collected and incubated for 20 min in cACSF at 37^°^C and additional 40 min in normal ACSF (ACSF) containing (in mM): 125 NaCl, 2.5 KCl, 1 MgSO_4_ × 7 H_2_O, 2 CaCl_2_ × H_2_O, 1.25 NaH_2_PO_4_ × H_2_O, 25 NaHCO_3_, and 25 d-glucose; pH: 7.4 (ingredients purchased from Carl Roth, Karlsruhe, Germany) at room temperature.

### 2.4. Multi-electrode array recordings

Spontaneous neuronal activity was recorded using a 2-chamber MEA system (MEA2100 System, Multi Channel Systems MCS GmbH, Kusterdingen, Germany). A chip consists of 60 electrodes (59 recording electrodes, 1 internal reference, 60MEA200/30iR; Multi Channel Systems MCS GmbH, Kusterdingen, Germany) with a distance of 200 μm between electrodes. The diameter of each recording electrode was 30 μm.

**FIGURE 1:**
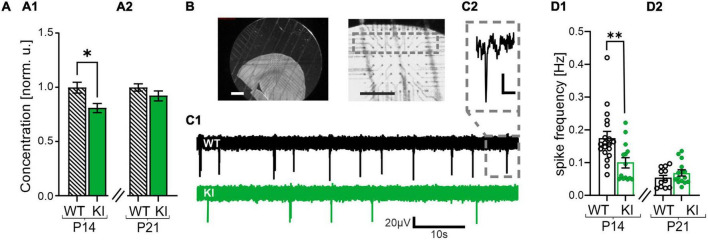
Reduced gamma-aminobutyric acid (GABA) concentration and impaired spontaneous network activity in somatosensory cortex of KI-mice. **(A)** GABA concentration in whole cortex lysates of P14 **(A1)** and P21 KI mice **(A2)** normalized to wild-type (WT) littermates and recorded by ELISA. Note the reduced GABA concentration in KI mice at P14. **(B)** Representative images of an acute cortical brain slice placed on the multi-electrode array (MEA) Chip (left and middle). The gray square represents the recording area, layers 2/3. Scale bars = 10 mm. **(C1)** Representative voltage traces of WT (above) and KI (below) mice at P14 recorded with the MEA. **(C2)** The inset shows a spike event at higher magnification, Scale bar = 20 μV/10 ms **(D1)** Mean spontaneous spike frequency in WT and KI mice at P14 and at P21 **(D2)**. Note the reduced frequency in P14 KI animals compared to WT littermates.

The cortical slices were placed on the chip in a way that the top border of the slice was aligned with the border of the top electrode row (see Figure 1B). Electrode rows two and three corresponded to cortical layers 2/3 were used for recordings and analysis. The location of the somatosensory cortex was identified according to the mouse brain atlas by Paxinos and Franklin (2004).

Slices were fixed on the MEA chip using a platinum grid and constantly perfused with oxygenated ACSF at a flow rate of 1.5 ml/min using a centrifugal pump (Gilson international, Berlin, Germany). The recording chamber containing ACSF was heated to 32^°^C. The slices equilibrated on the chip for 30 min before starting the electrophysiological recordings. Spontaneous activity was sampled at 25 kHz. The raw data were filtered using a Butterworth high-pass second order filter with a 200 Hz cut-off. The cortical spontaneous network activity was recorded for 5 min. Every event crossing the 5-fold standard deviation (SD) of the noise was considered as a spike. Spike detection was performed using Multichannel Analyzer 2.18 software (Multi Channel Systems MCS GmbH, Kusterdingen, Germany).

### 2.5. Whole-cell patch-clamp recordings

Brain slices were transferred into the recording chamber mounted on an upright microscope (Olympus, BX50WI, Shinjuku, Japan). The slices were constantly perfused with oxygenated ACSF using a gravity-driven system with a flow rate of 1.5 ml/min.

All electrophysiological signals were recorded using an Axopatch-200B amplifier and Clampex 11.2 software (Molecular Devices, San José, CA, USA). Pipettes were pulled using a DMZ Zeitz-Puller (Planegg, Germany). The resistance of the glass pipettes ranged between 3 and 10 MOhm when filled with the intra-pipette solution. Depending on the experiment, two different intracellular solutions were used. The potassium-based intracellular solution contained (in mM) K-gluconate, 140; KCl, 8; MgCl_2_ × 6 H_2_O, 2; Na_2_ATP, 4; Na_2_GTP hydrate, 0.3; Na_2_Phosphocreatin, 10; HEPES Potassium salt, 10. The cesium-based intracellular solution contained (in mM) Cs-gluconate, 125; CsCl, 5; EGTA, 10; MgCl_2_, 2; Na_2_ATP, 2; Na_2_GTP, 0.4; HEPES, 10; N-Ethyl lidocaine bromide (QX-314), 5. The pH was set to 7.3 by KOH and CsOH for K^+^- and Cs^+^-based solutions, respectively.

Slices were optically inspected with a low magnification objective (5X, Zeiss, Oberkochen, Germany) to identify the area of the somatosensory cortex. Next, individual pyramidal neurons were optically selected for the desired patch-clamp recordings in cortical layers 2/3 using a 40X objective (Olympus, Shinjuku, Japan). Recordings started at least 5 min after establishing the whole-cell configuration to allow the proper wash-in of the intra-pipette solution. For measurements in current-clamp mode the recorded pyramidal neurons were kept at their resting membrane potential and the K^+^-based intrapipette solution was used. We used a stimulation protocol with a pulse duration of 500 ms and gradually increasing current steps ranging from −20 to +480 pA. Input resistance and passive membrane properties were calculated from the hyperpolarization steps and applying a mono-exponential fit. The first action potential (AP) was used to analyze the functional properties of APs, including threshold, risetime and amplitude. The frequency of the APs was calculated from the maximum number of APs observed during a 500 ms depolarizing current step.

Both, miniature inhibitory postsynaptic currents (mIPSCs) and miniature excitatory postsynaptic currents (mEPSCs), were recorded under voltage clamp conditions with a cesium-based intrapipette solution. The bathing solution contained tetrodotoxin (TTX, 1 μM), a selective blocker of voltage-gated Na^+^ channels, and it contained 2-amino-5-phosphonovalerinans acid (DAP-5, 25 μM), an antagonist of NMDA receptors. The recorded neurons were initially voltage-clamped to a holding potential of −70 mV. For mEPSCs, the cells were kept at −60 mV, which is the reversal potential for GABA_A_-receptor-mediated currents. The mIPSCs were recorded at a holding potential of +10 mV, which is the reversal potential of AMPA-receptor-mediated currents. Serial resistance compensation was not applied. The mIPSC/mEPSC signals were recorded at least for 5 min. Signals were filtered at 3 kHz and sampled at 25 kHz. All electrophysiological data were analyzed using the Clampfit11 software (Molecular Devices, San José, CA, USA). The specific antagonists of GABA_B_Rs, CGP55845, and of GABA transporters GAT-3, SNAP5114, as well as the specific GABA_B_R-agonist Baclofen were provided by Tocris (Bio-Techne, Wiesbaden, Germany). All other chemicals were purchased by Carl Roth (Karlsruhe, Germany).

### 2.6. Statistics

All data were analyzed using Excel 2019 (Microsoft, USA) and GaphPad Prism software (San Diego, CA, USA). Results are presented as mean ± standard error of the mean (SEM). If not otherwise noted, a pairwise Mann-Whitney Test was performed for non-parametric distributions. For comparison of more than two experimental groups we performed a Kruskal-Wallis Test with Dunn’s multiple comparison (mentioned in the text). Statistical significant differences between experimental groups are displayed by asterisks *P < 0.05; ^**^P < 0.01; ^***^P < 0.001.

## 3. Results

### 3.1. GAD67-GFP KI mice show lower network activity compared to WT littermates

GAD67 is the main neuronal GABA synthesizing enzyme in early development (Greif et al., 1991). Haplodeficiency of this enzyme in the present KI-mouse model was reported to result in a reduction of GABA concentration in the whole brain (Tamamaki et al., 2003). First, we verified the previous finding of a reduced GABA concentration in this mouse model by performing a GABA ELISA experiment of whole brain lysates. As expected, we observed a reduced GABA concentration in the cortex of young KI mice at P14 compared to their aged-matched WT littermates (0.8 ± 0.04 in KI as compared with WT, *n* = 7, *p* = 0.0175, Figure 1A1). This reduction was no longer visible at the age of 21 days (0.9 ± 0.04 in KI as compared with WT, *n* = 7, *p* = 0.3829, Figure 1A2). Theoretically, such a reduction in the concentration of the main inhibitory neurotransmitter GABA at P14 should boost cortical network activity. We tested this hypothesis by performing MEA recordings from the somatosensory cortex and recording of spontaneous spiking frequency in the tissue (Figures 1B, C1, C2). To our surprise we observed a reduced spontaneous spiking frequency in cortical slices from P14 KI mice compared to their WT littermates (KI: 0.1 ± 0.02 Hz, *n* = 14; WT: 0.2 ± 0.02 Hz, *n* = 19, *p* = 0.0025; Figure 1D1). This effect was transient, since no significant differences were observed in the cortex from older mice at P21 (WT: 0.05 ± 0.01 Hz, *n* = 11; KI: 0.07 ± 0.01 Hz, *n* = 15, *p* = 0.413, Figure 1D2). The observed hypoactivity at P14 likely originates from a compensatory mechanism to protect cortical networks from hyperactivity that would arise from GABA-deficiency in the P14 KI mice. This mechanism might include intrinsic neuronal alterations like changes in passive or active membrane properties, and/or an altered network excitability mediated by changes in synaptic functions.

### 3.2. No changes in active and passive membrane properties between KI and WT mice

To investigate whether the observed differences in spontaneous network activities are caused by alterations of the intrinsic membrane properties, we performed whole-cell patch-clamp recordings from visually identified pyramidal neurons in layers 2/3 of the somatosensory cortex. The passive membrane properties revealed no significant changes between the genotypes (Supplementary Table 1). Next, we analyzed functional properties of evoked action potentials (APs), including amplitude, threshold and rise-time. Although the AP threshold and the maximal firing rate of APs were significantly altered between WT and KI mice at P21 (Supplementary Table 1), those findings cannot explain the reduced spontaneous network activity in KI mice at P14. So, we conclude that GAD67 haplodeficiency does not affect the passive and active membrane properties of pyramidal neurons to explain the altered spontaneous network activity in the somatosensory cortex at P14.

### 3.3. Altered basal synaptic transmission in KI mice caused an increased E/I ratio in P14, but not in P21 mice

Next, we focused on synaptic neurotransmission in the somatosensory cortex of the KI mice. We explored potential changes in excitatory and inhibitory synaptic currents and performed whole-cell patch-clamp experiments to recorded mEPSCs and mIPSCs in presence of 1 μM TTX, a blocker of voltage-gated Na^+^ channels, and 25 μM DAP-5, an antagonist of NMDA receptors. The cesium-based intracellular solution allowed us to exclusively record GABAergic mIPSCs at a holding potential of +10 mV as well as exclusively glutamatergic mEPSCs at holding potential of −60 mV from the same recorded neuron (see section “2 Materials and methods”).

**FIGURE 2:**
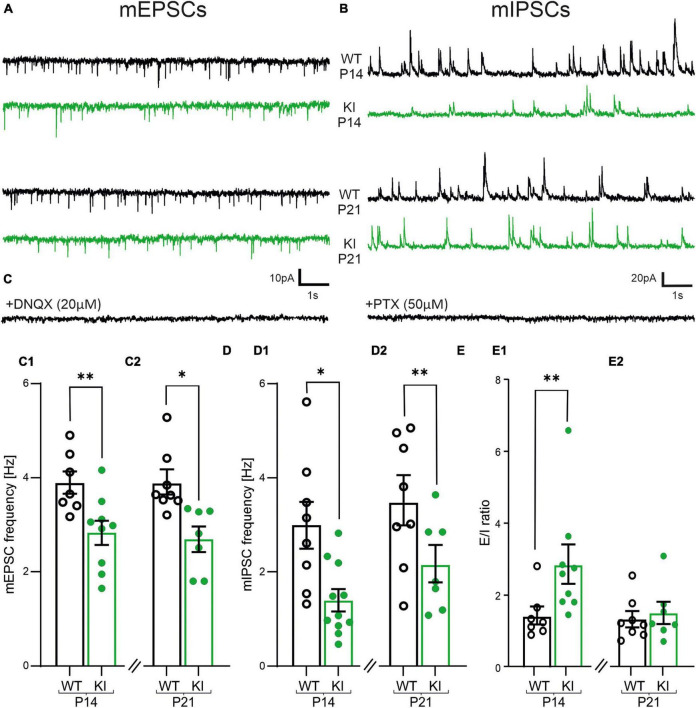
Impaired frequencies of miniature excitatory postsynaptic currents (mEPSCs) and miniature inhibitory postsynaptic currents (mIPSCs) and imbalanced E/I-ratio in layers 2/3 pyramidal cells of KI mice. **(A)** Representative traces of mEPSCs recorded at −60 mV in the presence of 25 μM DAP-5 and 1 μM TTX. DNQX (20 μM, bottom trace) completely abolished all inward currents confirming that they are AMPA-receptor dominated. **(B)** Representative traces of mIPSCs recorded at +10 mV from same neurons as shown in **(A)**. Picrotoxin (PTX, 50 μM, bottom trace) abolished all signals proving that they are originating from GABA_*A*_-receptor activity. **(C)** Summary graph showing the mean frequency of mEPSCs, which are reduced in P14 **(C1)** and P21 **(C2)** KI-mice. **(D)** The mean frequency of mIPSCs is also reduced in P14 **(D1)** and P21 **(D2)** KI-mice compared to WT littermates. **(E)** Summary graph of the ratio from the frequency of mEPSCs vs. mIPSCs, which is increased at P14 **(E1)**.

Interestingly, the glutamatergic, AMPA-receptor-mediated mEPSCs revealed a reduced frequency in KI mice compared to their littermates in both age groups (WT, P14: 3.9 ± 0.2 Hz, *n* = 7; KI, P14: 2.8 ± 0.3 Hz, *n* = 9, *p* = 0.0115; WT, P21: 3.9 ± 0.2 Hz, *n* = 8; KI, P21: 2.7 ± 0.2 Hz, *n* = 7, *p* = 0.0022, Figures 2A, C1, C2). Neither amplitudes nor kinetics of the mEPSCs were significantly different in KI and WT mice (Supplementary Table 2). As expected from the haplodeficiency of GAD67, the frequency of GABA_A_ receptor-mediated mIPSCs was also reduced in KI mice compared to WT littermates in both age groups (WT, P14: 3 ± 0.5 Hz, *n* = 8; KI, P14: 1.4 ± 0.2 Hz, *n* = 11, *p* = 0.0068; WT, P21: 3.5 ± 0.5 Hz, *n* = 8; KI, P21: 2.2 ± 0.3 Hz, *n* = 7, *p* = 0.0401, Figures 2B, D1, D2). Again, the amplitudes and kinetics of mIPSCs were comparable between WT and KI animals (Supplementary Table 2). Although both, excitatory and inhibitory synaptic inputs were reduced in KI mice, the E/I-ratio, defined as the ratio of the frequency of mEPSCs vs. mIPSC was significantly higher at P14 (WT, P14: 1.4 ± 0.2, *n* = 7; KI, P14: 2.8 ± 0.5, *n* = 9, *p* = 0.0079, Figure 2E1). Interestingly, this imbalance was transient and no longer visible at P21 (WT, P21: 1.3 ± 0.2, *n* = 8; KI, P21: 1.5 ± 0.3, *n* = 7, *p* = 0.7789, Figure 2E2). To ensure that the observed differences in mPSC frequencies were not biased by a lower detection of small-amplitude events, we also compared the 10% of events with the highest signal amplitude. The kinetics and amplitudes of these events were not different between genotypes (Supplementary Table 3), confirming the reduction only in frequencies of miniature postsynaptic currents. We conclude that the E/I ratio in the somatosensory cortex of KI mice at P14 is shifted toward stronger excitation (Figure 2E1), despite the overall reduced network activity (Figure 1D1). This on the first view contradictory finding suggests the existence of an additional mechanism to counterbalance the elevated E/I-ratio. GABA can activate both, ionotropic GABA_A_ receptors as well as metabotropic (inhibitory) GABA_B_R. We hypothesized that the latter one would be the key candidate to mediate the downscaling of neuronal network activity through ambient GABA.

### 3.4. GABA_B_Rs are tonically activated on glutamatergic presynapses in KI mice

**FIGURE 3:**
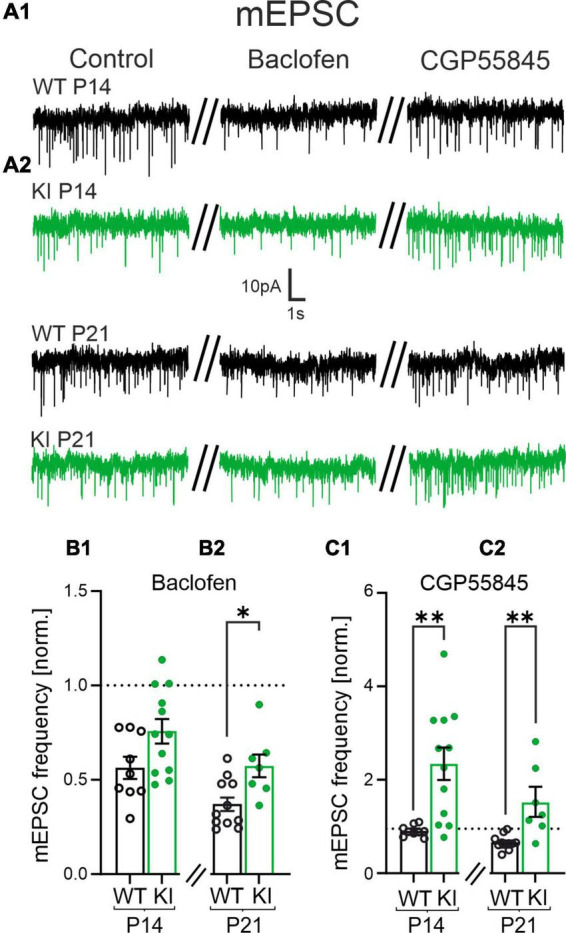
The reduced frequency of miniature excitatory postsynaptic currents (mEPSCs) is mediated by activity from presynaptic GABA_B_ receptors (GABA_B_Rs) at glutamatergic synapses. **(A)** Representative mEPSCs of P14 and P21 mice under control condition (left), in presence of Baclofen (10 μM, middle) and in CGP55845 (1 μM, right trace). **(B1,B2)** Summary diagrams of the relative mean frequency of mEPSCs in presence of the GABA_B_R agonist Baclofen normalized to the recordings made in control artificial cerebrospinal fluid (ACSF). **(B2)** Note the significantly smaller reduction in the frequency in wild-type (WT)-animals at P21. **(C1,C2)** The GABA_B_R- antagonist CGP55845 restored the mEPSC frequency back to control levels in WT mice at P14 and at P21. Note, CGP55845 strongly increased the mEPSC frequency in KI-mice above the level of the control data.

GABA_B_Rs are G_i_ protein-coupled receptors, which can modulate the frequency of both, mEPSCs and mIPSCs (Alten et al., 2022). To test the role GABA_B_Rs on the frequency of mEPSCs, we performed whole-cell patch-clamp recordings with a K^+^-based intracellular solution. First, we recorded the baseline mEPSCs in drug-free aCSF. Next, we bath-applied the GABA_B_R agonist Baclofen (10 μM) followed by the GABA_B_R antagonist CGP55845 (1 μM). Baclofen significantly decreased the frequency of mEPSCs in all experimental groups normalized to control conditions (Kruskal-Wallis Test with Dunn’s multiple comparison, *p* < 0.05), except for the P14 KI mice, which however, showed a similar trend (Figures 3A, B1, B2). Notably, Baclofen had a significantly weaker effect on KI mice tissue at the age of P21 compared to WT controls (WT, P14: 0.6 ± 0.06, *n* = 9; KI, P14: 0.8 ± 0.06, *n* = 12 *p* = 0.0585; WT, P21: 0.4 ± 0.04, *n* = 11; KI, P21: 0.6 ± 0.06, *n* = 7, *p* = 0.0165, Figure 3B2). This indicates that additional activation of GABA_B_Rs through baclofen is less effective at glutamatergic synapses of the KI mice at P14 and P21, suggesting that GABA_B_Rs were already tonically activated by ambient GABA.

Next, we tested if presynaptic GABA_B_Rs were indeed activated by ambient GABA. We bath applied the specific GABA_B_R-antagonist CGP55845 (1 μM) and observed no changes in the frequency of mEPSCs in WT mice. This suggested that presynaptic GABA_B_Rs at glutamatergic terminals of WT mice are not strongly activated by ambient GABA. In contrast, CGP55845 significantly increased the frequencies of mEPSCs in KI animals in both age groups (WT, P14: 1 ± 0.04-fold, *n* = 9; KI, P14: 2.5 ± 0.4-fold, *n* = 12, *p* = 0.0018; WT, P21: 0.7 ± 0.05-fold, *n* = 11; KI, P21: 1.6 ± 0.3-fold, *n* = 7, *p* = 0.003, Figures 3A1, A2, C1, C2). We also compared the absolute frequency of mEPSCs in the presence of CGP55845. At both age groups, we could no longer detect a significant difference of the mEPSCs frequency between genotypes in the presence of CGP55845 (*p* > 0.05). This suggested that presynaptic GABA_*B*_Rs are tonically activated in KI mice, thereby inhibiting the glutamatergic strength in the cortical network.

### 3.5. No tonic presynaptic inhibition at GABAergic synapses in KI mice

**FIGURE 4:**
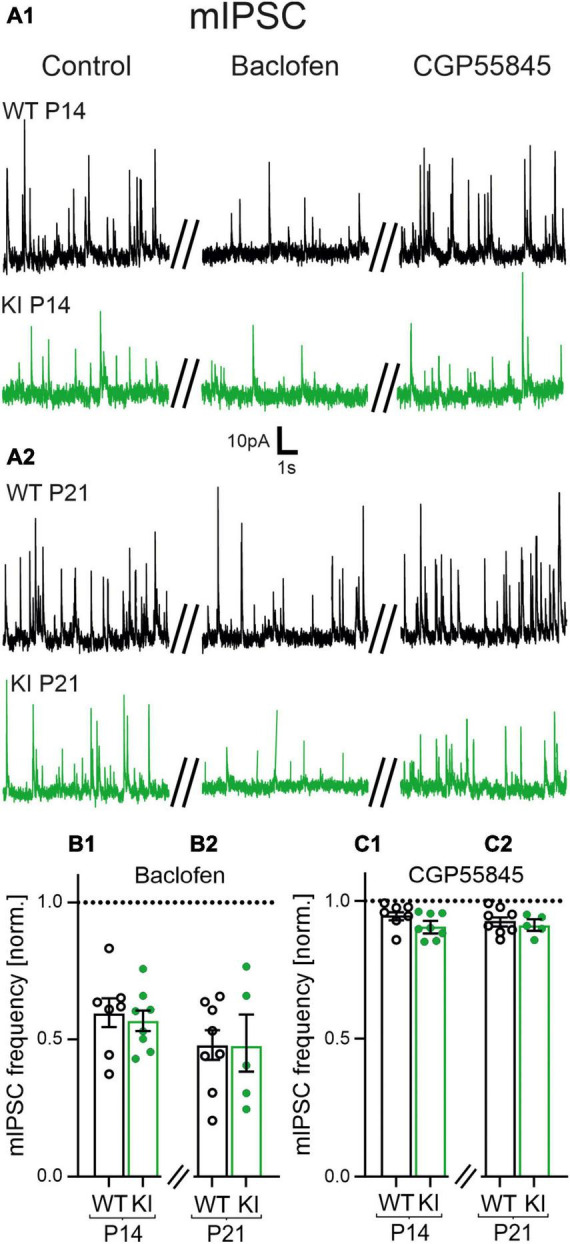
Role of GABA_B_ receptors (GABA_B_Rs) for the impaired frequency of miniature inhibitory postsynaptic currents (mIPSCs) at inhibitory synapses. **(A1,A2)** Representative current traces of mIPSCs of P14 and P21 mice under control condition (left), in the presence of Baclofen (10 μM, middle) and CGP55845 (1 μM, right). **(B1,B2)** Baclofen decreased the mean relative frequency of mIPSCs in all age groups and genotypes. Data were normalized to corresponding control signals in control artificial cerebrospinal fluid (ACSF). **(C1,C2)** CGP55845 rescued the frequency of mIPSCs back to the level of the corresponding control recordings in all age groups and genotypes.

Next, we performed similar experiments with the cesium-based intracellular solution to examine any presynaptic effect of GABA_B_Rs on GABAergic synapses in KI mice. Baclofen strongly reduced the frequency of mIPSCs compared to control conditions in both, WT and KI mice at both ages (Figures 4A1, A2) (Kruskal-Wallis Test with Dunn’s multiple comparison; *p* < 0.05). However, no significant difference was observed between the two genotypes (WT, P14: 0.6 ± 0.05, *n* = 7; KI, P14: 0.6 ± 0.04, *n* = 8, *p* = 0.6126; WT, P21: 0.5 ± 0.05, *n* = 8; KI, P21: 0.5 ± 0.09, *n* = 5, *p* = 0.9999, Figures 4B1, B2). Hence, GABAergic synapses express functional GABA_B_Rs independent of genotypes and age. Interestingly, and in contrast to the glutamatergic system, blockade of GABA_B_Rs with CGP55845 failed to increase the frequency of mIPSCs in KI mice of both age groups compared to their WT littermates (WT, P14: 1 ± 0.02, *n* = 7; KI, P14: 0.9 ± 0.01, *n* = 8, *p* = 0.0939; WT, P21: 0.9 ± 0.02, *n* = 8; KI, P21: 0.9 ± 0.01, *n* = 5, *p* = 0.6216, Figures 4C1, C2). To substantiate the results, we also compared the absolute frequency of mIPSCs in the presence of CGP55845. The mIPSC frequency was still significantly different between the two genotypes in both age groups (*p* < 0.05). This indicates that GABAergic synapses are functionally not affected by tonic inputs from presynaptic GABA_B_R. Together with the results on glutamatergic synapses we conclude that tonically activated GABA_B_Rs in KI mice inhibit the excitatory synaptic drive, thereby reducing network excitability.

### 3.6. Tonic activation of postsynaptic GABA_B_Rs in KI mice

**FIGURE 5:**
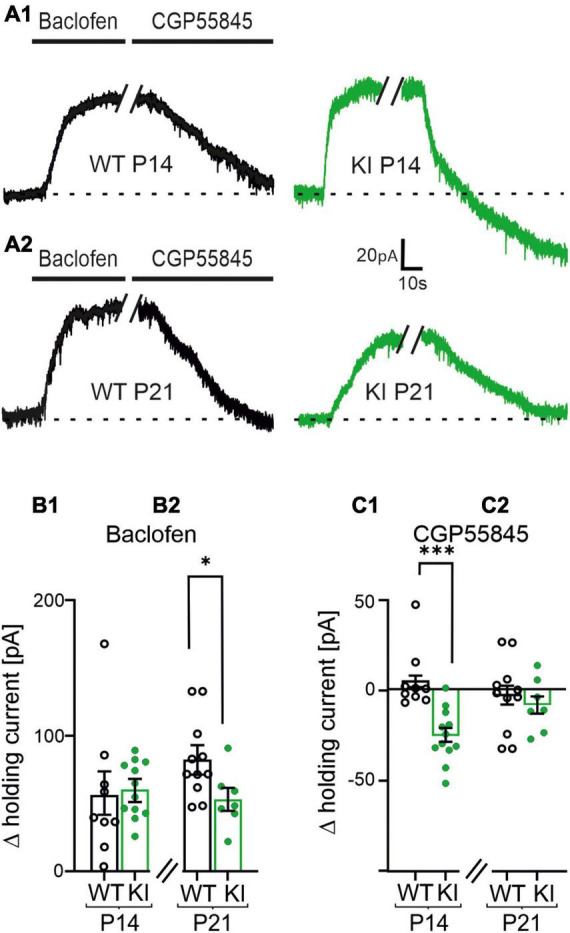
Postsynaptic GABA_B_ receptors (GABA_B_Rs) shift the holding current of layers 2/3 pyramidal cells during voltage-clamp recordings. **(A1,A2)** Representative current traces of voltage clamped pyramidal neurons at constant holding potential of −60 mV in P14 and P21 wild-type (WT)- and KI-mice under control conditions, in presence of Baclofen (10 μM) or CGP55845 (1 μM). **(B1,B2)** Summary diagram of the mean changes in holding current induced by Baclofen or CGP55845. Baclofen shifted the holding current corresponding to a hyperpolarization of the cells. Note, the hyperpolarization was significantly weaker expressed in P21 KI mice as compared to WT littermates **(B2)**. **(C1,C2)** Effect of CGP55845 on the shift of the holding current. The GABA_B_R-antagonist reversed the effect of Baclofen. Importantly, CGP55845 induced a current shift, which even corresponds to a depolarization of the cells in P14 KI mice.

Postsynaptic GABA_B_Rs can also affect neuronal signaling (Tao et al., 2013; Gerrard et al., 2018; Shaye et al., 2021; Bassetti, 2022). Therefore, we investigated a potential contribution of postsynaptic GABA_B_Rs in our mouse model. First, we performed whole-cell patch clamp experiments to disclose changes in the holding current of pyramidal neurons induced by Baclofen or CGP55845. We used a K^+^-based intracellular solution and voltage-clamped the neurons to a holding potential of −60 mV. The GABA_B_R agonist Baclofen shifted the holding current toward a hyperpolarization in both genotypes, indicating expression of functional GABA_B_Rs at the postsynaptic site. The Baclofen-induced shift was not different between KI and WT mice at P14 (WT, P14: 56 ± 15 pA, *n* = 9; KI, P14: 60 ± 6 pA, *n* = 12 *p* = 0.31; Figures 5A1, B1), however, it was smaller in KI animals at P21 compared to their age matched littermates (WT, P21: 82 ± 8 pA, *n* = 11; KI, P21: 53 ± 7 pA, *n* = 7, *p* = 0.0285, Figures 5A2, B2). The GABA_B_R-antagonist CGP55845 reversed the effects of Baclofen in WT mice at both ages and in KI mice at P21. Notably, CGP55845 induced a significant shift of the holding current toward depolarization at P14 in the KI mice (Figure 5A1). This indicates an existence of tonically activated GABA_B_Rs at the postsynaptic site of P14 KI (WT, P14: 5 ± 5 pA, *n* = 9; KI, P14: −26 ± 4 pA, *n* = 12 *p* = 0.0002; WT, P21: −4 ± 6 pA, *n* = 11; KI, P21: 8 ± −5 pA, *n* = 7, *p* = 0.6119, Figures 5A1, A2, C1, C2). Together, tonic GABA_B_R-mediated postsynaptic inhibition in concert with stronger tonic presynaptic inhibition at glutamatergic synapses could explain the overall reduction of network excitability in the cortex of KI mice at P14. These data also provide a possible explanation for the compensation of the network activity at P21 (see Figure 1). The loss of the tonic postsynaptic GABA_B_R-mediated inhibition in the P21 KI can lead to compensation of the network activity at P21.

### 3.7. Tonic GABA_B_R activation mediated decreased network activity in KI mice

**FIGURE 6:**
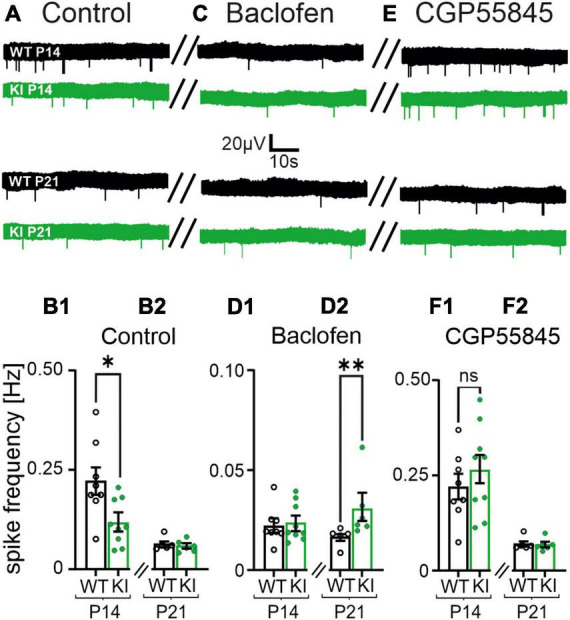
Spontaneous cortical network activity of acute coronal brain slices is affected by Baclofen and CGP55845. **(A)** Representative voltage traces from multi-electrode array (MEA) recordings under control conditions in P14 and P21 mice (left column traces). **(B1,B2)** The mean spike frequency is impaired in KI mice at P14 in normal artificial cerebrospinal fluid (ACSF). **(C)** Representative voltage traces in presence of Baclofen (10 μM, middle column). **(D1,D2)** Baclofen further reduced the mean spike frequencies. Note, the impairment is weaker in KI mice at P21 compared to wild-type (WT) littermates. **(E)** Representative voltage traces in presence of CGP55845 (1 μM). **(F1,F2)** CGP55845 rescued the level of spontaneous activity back to control levels in P21 mice. Note the spike frequencies are no longer significantly different between KI and WT mice at P14.

To further verify the proposed mechanism, we measured cortical spontaneous network activity in presence of Baclofen (10 μM) or CGP55845 (1 μM) in MEA recordings of acute slice. As already shown in Figure 1, the spontaneous firing frequency was impaired in KI-mice at P14 compared to WT-controls (WT, P14: 0.2 ± 0.03 Hz, *n* = 8; KI, P14: 0.1 ± 0.02, *n* = 9, *p* = 0.0206; Figure 6B1). In presence of the GABA_B_R-agonist we could not observe differences in spontaneous network activity between KI and WT mice at P14 (Figure 6D1). However, Baclofen had a significantly weaker effect on the cortical activity in KI-mice at P21 when compared to WT-animals of the same age (WT, P21: 0.02 ± 0.002 Hz, *n* = 5; KI, P21: 0.03 ± 0.005 Hz, *n* = 6, *p* = 0.0087; Figure 6D2). This indicates an already activation of GABA_B_-Rs in KI-animals. In presence of the GABA_B_R-blocker we observed no significant difference in spontaneous activity between KI and WT mice in both age groups (WT, P14: 0.2 ± 0.03 Hz, *n* = 8; KI, P14: 0.3 ± 0.04 Hz, *n* = 9, *p* = 0.3213; WT, P21: 0.07 ± 0.007 Hz, *n* = 5; KI, P21: 0.07 ± 0.006, *n* = 6, *p* = 0.8896; Figures 6F1, F2). In summary, the increased tonic activation of GABA_B_Rs in the cortex of KI mice at P14 mediated the reduction of spontaneous network activity, despite the reduction in total amount of GABA and the observed shift of E/I ratio toward excitation.

### 3.8. GAT-3 provides GABA for the tonic GABA_B_R activation

The haplodeficiency in GAD67 in our KI-mice leads to a reduced neuronal GABA synthesis, so we speculated on alternative main sources for the generation of the ambient GABA in these animals. Our experiments so far provided evidence that ambient GABA activates GABA_B_Rs on glutamatergic pre- and post-synapses to compensate for the impaired GABAergic inhibition. In the cerebral cortex the GABA transporter 3 (GAT-3) has been shown to operate in reverse mode, i.e., releasing GABA, under resting conditions (Kinney, 2005). As GAT-3 is predominantly localized at astrocytes (Minelli et al., 1996) and GAD67 expression in astrocytes is very low (Schousboe et al., 1992), we hypothesized that a reduction of neuronal GAD67-mediated GABA synthesis may facilitate GABA efflux via GAT-3.

**FIGURE 7:**
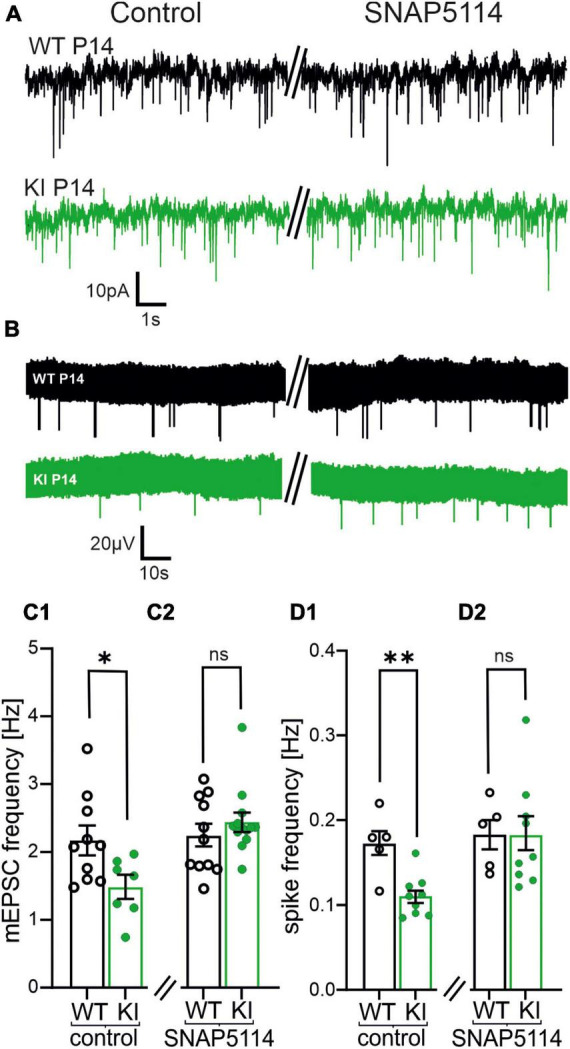
Blockade of the astrocytic transporter GABA transporter 3 (GAT-3) increases both, frequency of miniature excitatory postsynaptic currents (mEPSCs) and spontaneous activity in KI mice at P14. **(A)** Representative traces of mEPSCs in controls (left) and after incubation of SNAP5114 (50 μM, right). **(B)** Representative voltage traces of spontaneous activity by multi-electrode array (MEA) recordings under control conditions (left) and after incubation with SNAP5114 (right). **(C1,C2)** Summary diagram of the mean frequency of mEPSCs in KI and WI mice in controls and in the presence of SNAP5114. Note that the mEPSC frequency recovers to a level similar to wild-type (WT)-mice in presence of the GABA transporter 3 (GAT-3)-blocker in KI animals **(C2)**. **(D1,D2)** Summary diagram of the mean spontaneous activity in the cortical slice in KI and WI mice in controls and in presence of SNAP5114. Also, the spontaneous activity increases to the level of WT-mice in SNAP5114 **(D2)**.

To test this hypothesis, we recorded mEPSCs using a potassium K^+^-based intracellular solution (Figures 7A, C1). Then we incubated the slices with SNAP5114 (50 μM), a selective GAT-3 blocker. In presence of the GAT-3 blocker no differences in the frequencies of mEPSCs were observed in KI- compared to WT-mice (WT, P14: 2.2 ± 0.2 Hz, *n* = 11; KI, P14: 2.44 ± 0.13 Hz, *n* = 13, *p* = 0.5029, Figures 7A, C2). To corroborate this result, we also performed MEA recordings from P14 cortical slices (Figures 7B, D1). Similar to the effects on mEPSCs, SNAP-5114 led to a comparable spontaneous network activity in KI mice and WT littermates (WT, P14: 0.2 ± 0.02 Hz, *n* = 5; KI, P14: 0.2 ± 0.02, *n* = 9, *p* = 0.7972; Figures 7B, D2). Finally, we tested whether GAT-3-mediated GABA efflux was able to evoke a tonic activation of GABA_B_Rs. Indeed, in presence of the GAT-3 blocker, any GABA_B_R inhibition through CGP55845 failed to affect the frequency of mEPSCs (WT, P14: 2.5 ± 0.2 Hz, *n* = 4; KI, P14: 2.6 ± 0.1 Hz, *n* = 4, *p* = 0.4857; Supplementary Figure 1). These results suggest that GAT-3 mediated efflux is the main source of extracellular GABA under these conditions, and GABA efflux prevented any cortical hyperactivity in the brains of young KI mice at P14.

## 4. Discussion

### 4.1. Transient reduction of GABA level in KI mice

The present heterozygous GAD67 (GFP)-mouse line (KI) was previously shown to express reduced concentrations of cortical GABA at P14 (Tamamaki et al., 2003). GABA levels in the present study were measured in whole cortex lysates using ELISA techniques, which does not allow to delineate changes in [GABA]_i_ in a cell type-specific manner. However, as GABA in the CNS is mostly located in GABAergic interneurons (Del Rio et al., 1992), our ELISA data suggested a low GABA availability and hence low GABAergic synaptic function in cortical networks of KI-mice. Indeed, the frequency of mIPSCs was significantly reduced in KI mice, indicating a weaker phasic GABAergic drive. Notably, weakening of inhibitory drive was not reflected by an increase in spiking activity of our acute coronal brain slices (Figure 1). Also, the intrinsic membrane and firing properties of pyramidal neurons were not altered between WT- and KI- mice. Therefore, we focused on potential functional changes in synaptic neurotransmission.

### 4.2. Changes of basal glutamatergic and GABAergic synaptic transmission

The frequency of mIPSCs as well as mEPSCs were significantly reduced in KI cortex. Reduction in frequency of mIPSCs (Figure 2) is in good agreement with previous reports on mouse models with a reduced GABA synthesis (Lau and Murthy, 2012; Lazarus et al., 2015). One might expect that this reduction of GABAergic inhibition would lead to exaggerated excitatory glutamatergic activity in the network. Instead, we also observed a reduction in the frequency of glutamatergic mEPSCs in the KI animals.

Low synaptic levels of GABA might also reduce filling of presynaptic vesicles with GABA (Zhou et al., 2000; Engel et al., 2001) and hence reduce the amplitudes of mIPSCs. Indeed, this has been reported for homozygous GAD1 knockout mice models by others (Lau and Murthy, 2012). However, the mIPSC amplitudes in our recordings were not different in KI-animals at P14 or at P21 (Supplementary Table 2). The differences likely arise from use of homozygous tissue plus cell cultures or slice cultures, in which the synaptic connectivity is likely to be altered as compared to our acute slice model from hemizygous GAD67 KI mice. Another possible explanation could be a compensatory upregulation of postsynaptic GABA_A_ receptors. In this case, we would also not see changes in the amplitude of mIPSCs.

Alterations in the subunit composition of postsynaptic receptors is known to be a possible postsynaptic compensatory mechanism to adjust synaptic strength (charge transfer). For example, this has been shown in neurodevelopmental disorders (Han et al., 2014; Hammer et al., 2015) (for review see: Kirischuk, 2022). In our KI-mouse model we did not observe significant differences in amplitude or kinetics of both, mIPSCs and mEPSCs between the genotypes (Supplementary Table 2). Consequently, changes in subunit composition of postsynaptic AMPA- as well as of GABA_A_ receptors are unlikely causing the reduced network activity in our KI mouse model.

### 4.3. Elevated E/I ratio associated with a reduced network activity

The reduced frequency of both mEPSCs and mIPSCs can affect the E/I ratio in KI-mice. An E/I imbalance was earlier observed in several animal models of neurological disorders (Rubenstein and Merzenich, 2003; Ghatak et al., 2021). Indeed, we also detected an increased E/I ratio in the KI mice at P14, but not at P21. This transient shift of E/I ratio toward more excitation resulted from a relatively stronger reduction in the inhibitory drive of KI mice (Figure 2). The frequency of mIPSCs increased with maturation from P14 to P21 in KI mice, thereby stabilizing the E/I ratio to WT levels at P21. One reason for this recovery might be that during maturation an additional GABA-synthesizing enzyme, GAD-65, is functionally expressed in synapses of neurons (Kiser et al., 1998) thereby increasing the level of GABA in the cortex.

Theoretically, the observed shift in E/I balance toward excitation should promote hyperactivity in the cortical networks. However, we observed a decrease in neuronal network activity (Figure 1). This is likely a result of an additional compensatory mechanism in the cortex of KI-mice to counterbalance the potential hyperexcitability. Elevated E/I ratios in cortical networks were also observed in various genetic mouse models of autism, which was also interpreted as a homeostatic stabilization mechanism of synaptic drive rather than driving network hyperexcitability (Antoine et al., 2019). Altered GABAergic functions were also observed during visual processing in humans suffering from ASD, and they were modulated by GABA_B_R activity (Huang et al., 2022). In a tumor suppressor gene 2 (Tsc2±) mouse ASD model both, the tonic pre- and postsynaptic GABA_B_Rs were shown to modulate the E/I ratio (Bassetti et al., 2020). However, so far the present KI mouse model was never reported to show an ASD-like behavior, which indicates that at least one additional cellular an mechanism is required to develop ASD phenotype.

### 4.4. Role of presynaptic GABA_B_Rs on basal synaptic transmission

GABA_B_ receptors are already expressed in the embryonic cortex (Turgeon and Albin, 1994). They fulfill trophic functions during early development as well as phasic and tonic synaptic transmission in mature brain (for recent review see: Bassetti, 2022). As GABA_B_Rs are metabotropic Gi-coupled receptors, their activation leads to a suppression of the adenylate cyclase as well as to the opening of specific K^+^ channels (GIRK-channels) and to the inhibition of presynaptic Ca^2+^ channels (for review: Padgett and Slesinger, 2010). In line with these actions of GABA_B_Rs we observed a reduction in frequency of glutamatergic mEPSCs in presence of the GABA_B_R agonist in all experimental groups. Blockade of GABA_B_Rs with CGP55845 exclusively increased the frequency of mEPSCs in KI mice, which is strong evidence that glutamatergic presynapses in KI-mice tissue are tonically inhibited by ambient GABA. Our data indicate that GABAergic synapses also express functional GABA_B_Rs in both genotypes and age groups. However, the blockade of GABA_B_Rs had no effect on the mIPSC frequency indicating that the receptors are not activated by ambient GABA. From these experiments we conclude that the decreased mIPSC frequencies in KI mice are most likely a consequence of reduced intracellular GABA concentration, and this might lead to a decreased number of presynaptic vesicles, thereby reducing the readily releasable pool (RRP).

### 4.5. GABA_B_R-mediated postsynaptic inhibition

Baclofen increased the holding current of pyramidal neurons in all experimental groups confirming others that pyramidal neurons express functional postsynaptic GABA_B_Rs (Terunuma et al., 2014). Interestingly, when GABA_B_Rs were blocked with GGP55845, we observed a significant depolarization in the KI mice tissue only at P14, suggesting tonic activation of postsynaptic GABA_B_Rs in mice with a haploinsufficiency for GAD67 at P14, but not in WT mice. It should be noted that although our data indicated GABA_B_R activation at the post-synapse of KI-mice at P14, we could not detect any changes in resting membrane potential or input resistance in these neurons. Still, this may be a result from the cellular localization of the GABA_B_R located directly on dendrites of the neurons (Booker et al., 2013).

**FIGURE 8:**
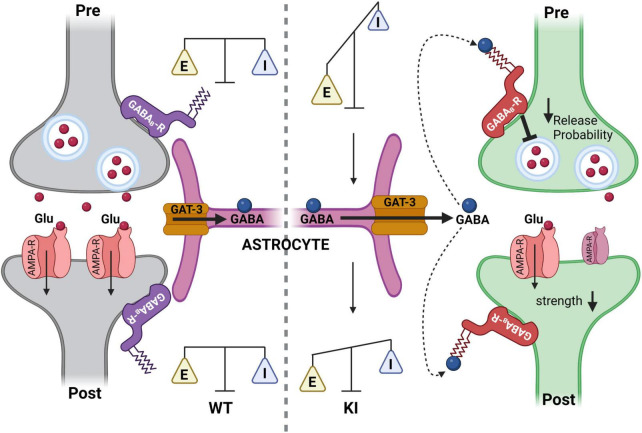
Summary cartoon illustrating the mechanism to counterbalance a temporal neuronal gamma-aminobutyric acid (GABA) deficit in KI mice at P14 through activation of tonic pre- and postsynaptic GABAB receptors (GABA_B_Rs) at glutamatergic synapses in the somatosensory cortex. **(Left)** Glutamatergic synapse of the somatosensory cortex in a wild-type (WT)-mouse at P14. GABA is taken up normally via GAT1 into GABAergic neurons (not shown) and via GABA transporter 3 (GAT-3) (orange) into astrocytes (purple). Subsequently, GABA_B_Rs (blue) are not activated. **(Right)** Glutamatergic synapse of the somatosensory cortex in a KI mice at P14. Astrocytes express the GAT-3 transporter, which is operating in reverse mode. The extrasynaptic GABA, released via GAT-3, can activate pre- and postsynaptic GABA_B_Rs. This activation of GABA_B_Rs leads to a decreased presynaptic glutamate release and decreased postsynaptic strength to counterbalance network activity, thereby preventing hyperexcitability in a GABA-deficient cortex (Figure created with Biorender.com).

So far, the data suggested the following cellular model (Figure 8) to balance the cortical network in KI-mice at P14: GABA_B_Rs were tonically activated by ambient GABA in mice with a haplodeficiency of GAD67 at P14. This led to a reduction in strength of presynaptic glutamatergic inputs. In parallel, tonically activated GABA_B_Rs on the postsynaptic site also functionally inhibited the excitatory pyramidal neurons. This might act as a compensatory mechanism to shift the E/I balance toward inhibition, thereby protecting the cortex from network hyperactivity. Tonic GABA_B_R activation is no longer present at the post-synapse at P21 (Figure 5) and shows at least a trend of reduction in the pre-synapse (Figure 3). Both results can be mediated by a negative feedback mechanism, which might occur due to the increased tonic activation of GABA_B_Rs at P14. This could lead to internalization of GABA_B_Rs, which impairs their impact on neuronal activity.

### 4.6. GABA_B_R dependent network activity

Tonically activated GABA_B_Rs can reduce the cortical network activity (see Figure 6). The slightly higher spontaneous activity under the presence of Baclofen in slices of KI at P21 could result from a reduced expression of functional GABA_B_Rs in pyramidal neurons at this age. This would agree with the theory raised above, that in KI at P21 GABA_B_R have a decreased influence on the neuronal activity. When all GABA_B_Rs were blocked with CGP55845, we could not observe any changes between the experimental groups. This indicated that on the network level tonically activated GABA_B_Rs stabilize the E/I ratio of single neurons at the cost of a decreased network activity, even in case of a reduced GABA synthesis like in the KI mouse. This raises the question of the putative GABA source.

### 4.7. GAT-3-mediated GABA release

In hippocampus, synaptically released GABA is the main source of extracellular GABA [GABA]_e_. GAT1 operating in uptake mode is the dominant transporter that keeps [GABA]_e_ low and prevents activation of extrasynaptic GABA_A_ and GABA_B_Rs (Jensen et al., 2003; Glykys and Mody, 2007). In the cerebral cortex however, GAT-3-mediated GABA efflux can be observed even under resting conditions (Kinney, 2005; Kirmse and Kirischuk, 2006). GATs transport one GABA molecule with one Cl^–^ and two Na^+^ ions (Attwell et al., 1993; Kristensen et al., 2011). As GABA is predominantly uncharged under physiological pH, the transport process is electrogenic and depends on three transmembrane gradients: Cl^–^, Na^+^ and GABA. The reduced GABAergic synaptic activity in the KI mice suggests a lower [GABA]_e_ and higher transmembrane GABA gradient. The latter might facilitate the observed tonic GAT-3-mediated GABA efflux in the cerebral cortex (Kinney, 2005), and is compatible with our observation: blockade of GAT3 shifted both, the frequency of mEPSCs and the network spiking activity back to the level of WT-controls. Moreover, when the GABA_B_R antagonist CGP55845 was applied on top of the GAT-3 inhibitor SNAP-5114, it did not affect the neuronal activity, confirming that GAT-3-mediated GABA release is the main mechanism that sets the strength of GABA_B_R-mediated inhibition in the cortex of KI mice. Interestingly, the GAT3 mediated increase in GABA level did not activate GABA_B_ receptors at inhibitory terminals. As an explanation for this we cannot exclude that GABAergic terminals express modified GABA_B_Rs, which are less-sensitive to GABA. However, since extracellular GABA is likely released via astrocytic GAT3, we propose that the spatial distribution of GAT3 and/or intra-astrocytic signaling, for example through changes in Na^+^ concentration, underlies the uneven activation of GABA_B_Rs.

## 5. Conclusion

Reduction of phasic GABA release in the KI mice is compensated by non-synaptic GAT-3-mediated GABA release. This leads to tonic activation of presynaptic GABA_B_Rs selectively at glutamatergic synapses, and in turn protects against cortical hyperactivity in this mouse model of neuronal GABA-deficiency. While GABA transporters like GAT-1 are localized predominantly on neurons, GAT-3 is expressed by astrocytes (Minelli et al., 1995; Minelli et al., 1996). GAT-3-mediated GABA release from astrocytes has been shown in several brain regions (for review see: Kilb and Kirischuk, 2022), and it was shown to have neuroprotective effects in animal models of brain injury (Cho et al., 2022). Elevation of intra-astrocytic Na^+^ leads to switching GAT-3 into GABA efflux mode (Heja et al., 2009; Heja et al., 2012). This mechanism could explain an increased extrasynaptic GABA release in the present KI mice model, since the increased E/I ratio at P14 would likely lead to stronger glutamate uptake by astrocytes, which in turn would facilitate GABA release. In addition, astrocytes are capable to synthesize GABA in a GAD67 independent manner from a monoamine putrescine (Jo et al., 2014; Kwak et al., 2020). It is tempting to speculate that the reduced neuronal GABA level in GAD67-GFP mice may be detected by astrocytes, resulting in facilitated GABA synthesis via GAD67-independent pathways resulting in a stronger non-synaptic GABA release. However, further investigations are required to verify this hypothesis.

## Data availability statement

The raw data supporting the conclusions of this article will be made available by the authors, without undue reservation.

## Ethics statement

All animal experiments conducted in this study were in accordance with national and European laws for the use of animals in research (2010/63/EU) and were approved by the local ethical committee.

## Author contributions

IT, SK, and TM planned the research. TU and CS performed and analyzed the experiments. TU, CS, IT, SK, and TM wrote the manuscript. All authors have read and approved the final manuscript.
