# Supplementary material for: Tonic activation of GABAB receptors via GAT-3 mediated GABA release reduces network activity in the developing somatosensory cortex in GAD67-GFP mice
Source: Front Synaptic Neurosci. 2023 May 30;15:1198159. doi: 10.3389/fnsyn.2023.1198159 (PMC10267986; doi:10.3389/fnsyn.2023.1198159)
Supplement: Supplementary file 1 [file Data_Sheet_1.docx]

Supplementary Material

**Tonic activation of GABA_B_ receptors via GAT3-mediated GABA release reduces network activity in the developing somatosensory cortex in GAD67-GFP mice**

Timo Ueberbach, Clara A. Simacek, Irmgard Tegeder, Sergei Kirischuk, Thomas Mittmann*

*** Correspondence:** Prof. Thomas Mittmann: mittmann@uni-mainz.de

## Table 1: Active and passive membrane properties of pyramidal cells do not differ between GAD67-GFP positive mice and littermates

Recordings from layers 2/3 pyramidal neurons in current-clamp mode. Passive Membrane Properties: Resting Membrane Potential (RMP) was read from the amplifier, Rm and Cm were calculated from hyperpolarizing steps using a monoexponential fit. Active Membrane Properties: Threshold, Amplitude and Risetime were calculated from the first action potential (AP) occurring. The frequency was calculated from the maximal number of APs observed during the depolarization steps. At P21, Threshold (WT: 29.6 ± 1.1 mV, n = 8; KI: 34.4 ± 1.3 mV, n = 7, p = 0.0312) and Frequency (WT: 24 ± 1 mV, n = 8; KI: 29 ± 1 mV, n = 7, p = 0.004, p=0,0151) were significantly different.

|  | **P14** | | **P21** | |
| --- | --- | --- | --- | --- |
|  | **WT (n=7)** | **KI (n=7)** | **WT (n=8)** | **KI (n=7)** |
| **Passive Membrane Properties** | | | | |
| RMP [mV] | 81.9 ± 1 | 80.9 ± 1.2 | 85.6 ± 1.2 | 84 ± 1.9 |
| Rm [mΩ] | 103.1 ± 5.5 | 97.9 ± 7.2 | 55.7 ± 4.4 | 48.3 ± 6.8 |
| Cm [pF] | 85 ± 6.7 | 79.5 ± 6.3 | 113 ± 5 | 98,8 ± 10.6 |
| **Active Membrane Properties** | | | | |
| Threshold [mV] | 30.2 ± 0.7 | 32.6 ± 1.5 | **29.6 ± 1.1** | **34.4 ± 1.3*** |
| Amplitude [mV] | 99.2 ± 3.7 | 96.8 ± 2.2 | 90.3 ± 1.1 | 92.2 ± 1.5 |
| Risetime [ms] | 1 ± 0.04 | 1 ± 0.04 | 0.9 ± 0.07 | 1,1 ± 0.08 |
| Frequency [Hz] | 25 ± 4 | 27 ± 2 | **24 ± 1** | 29 ± 1* |

## 1.2 Table 2: AMPA- and GABA-current properties

Recordings from layers 2/3 pyramidal neurons in voltage-clamp mode. Area and Decay Slope were calculated using a monoexponential fit.

|  | **P14** | | **P21** | |
| --- | --- | --- | --- | --- |
|  | **WT** | **KI** | **WT** | **KI** |
| **AMPA mediated currents** | | | | |
| Amplitude [pA] | 8.9 ± 1 | 11.1 ± 1.8 | 8.6 ± 0.7 | 8 ± 0.7 |
| Area [ms*pA] | 73.3 ± 8.8 | 81.7 ± 7.3 | 67.1 ± 3.6 | 64 ± 3.9 |
| Decay Slope [pA/ms] | 0.9 ± 0.1 | 1.1 ± 0.1 | 0.7 ± 0.1 | 0.7 ± 0.1 |
| **GABA mediated currents** | | | | |
| Amplitude [pA] | 18.4 ± 1.3 | 16.7 ± 1.3 | 14.7 ± 0.9 | 12.3 ± 1.4 |
| Area [ms*pA] | 354.6 ± 19.2 | 316.5 ± 17.3 | 307.9 ± 11.8 | 278.1 ± 27.6 |
| Decay Slope [pA/ms] | 0.4 ± 0.02 | 0.3 ± 0.02 | 0.4± 0,02 | 0.4± 0,02 |

## 1.3 Table 3: Top 10% AMPA- and GABA-current properties

Recordings from layers 2/3 pyramidal neurons in voltage-clamp mode. Area and Decay Slope were calculated using a monoexponential fit. Events were sorted due to their amplitude. All events with the top 10% in each recording were used for analyses. No significant difference in these events could be observed.

|  | **P14 (top10%)** | |
| --- | --- | --- |
|  | **WT** | **KI** |
| **AMPA mediated currents** | | |
| Amplitude [pA] | 19.9 ± 3.2 | 23.5 ± 2.5 |
| Area [ms*pA] | 158.2 ± 32.6 | 170.3 ± 18.6 |
| Decay Slope [pA/ms] | 1.5 ± 0.4 | 2.6 ± 0.6 |
| **GABA mediated currents** | | |
| Amplitude [pA] | 41.1 ± 5.3 | 36.3 ± 2.8 |
| Area [ms*pA] | 969 ± 100.8 | 786.7 ± 90 |
| Decay Slope [pA/ms] | 0.7 ± 0.07 | 0.6± 0.03 |

## 1.4 Supplementary Figure 1


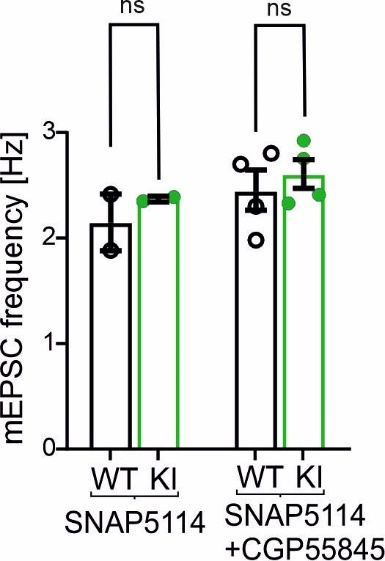


**Supplementary Figure 1.** mEPSC recordings from layers 2/3 pyramidal neurons in voltage-clamp mode. After the incubation with SNAP5114 (a GAT-3 blocker) the bath application of CGP55845 (a GABA_B_R blocker) had no significant influence on the mEPSC frequency.
